# Development of a psychological health promotion intervention for ultra-orthodox Jewish mothers of children with ADHD using the intervention mapping protocol

**DOI:** 10.1186/s12889-024-18126-4

**Published:** 2024-02-29

**Authors:** Jennifer R. Budman, Adina Maeir

**Affiliations:** School of Occupational Therapy, Faculty of Medicine of the Hebrew University, Mt. Scopus, P.O.B: 24026, Jerusalem, Israel

**Keywords:** Health promotion, ADHD, Intervention mapping, Mixed methodology

## Abstract

**Background:**

Attention Deficit Hyperactivity Disorder (ADHD) is a common neuro-developmental health condition in children and adolescents, in which its associated behavior manifestations are known to negatively affect members of the family unit, especially mothers. Ultra-orthodox Jewish (UOJ) community is growing globally and mothers of children with ADHD in this community are potentially at risk for negative health outcomes. As the UOJ community is culturally conservative, maintaining a distinct separation from outside influences, they often avoid utilizing public mental health services due to stigma and a lack of culturally sensitive treatments. Thus, this study aimed to develop a theory-driven and culturally appropriate psychological health promotion intervention for these mothers using the Intervention Mapping protocol.

**Methods:**

A mixed-method design was used. Qualitative content analysis was performed on four focus groups (*n*=25). Additionally, descriptive statistics including the content validity index was used to measure feedback regarding the developed intervention protocol’s relevance, effectiveness, and appropriateness Theoretical models for behavior change, including the Behavior Change Wheel’s COM-B system and the Theoretical Domains Framework, and literature on ADHD in the general population and the ultra-orthodox community were integrated in the process. Intervention components were systematically derived from findings.

**Results:**

Key determinants of health behavior change were identified, resulting in formulating intervention objectives addressing stigma reduction surrounding ADHD, increasing knowledge about the ADHD condition and treatment, awareness of the school systems’ capabilities in meeting the ADHD child’s needs, enhancing mothers’ advocacy skills, and maternal self-care. Intervention? strategies included a group setting, providing information on health consequences, social support, re-attribution, active learning, goal setting, and promoting an identity associated with the desired behavior change. Mothers’ quantitative feedback confirmed the overall relevance, effectiveness, and appropriateness of the interventions’ content (CVI^avg^= .86, .85, .87).

**Conclusions:**

Intervention Mapping facilitated the development of a culturally sensitive psychological health promotion intervention for ultra-orthodox Jewish mothers of children with ADHD. Further research is warranted to assess intervention feasibility and effectiveness.

**Supplementary Information:**

The online version contains supplementary material available at 10.1186/s12889-024-18126-4.

## Background

Attention Deficit Hyperactivity Disorder (ADHD) is a chronic neurodevelopmental health condition characterized by persistent symptoms of inattention, hyperactivity, and impulsivity [1]. It affects approximately 5-7% of children worldwide, making it one of the most prevalent psychiatric disorders among this age group [2]. While ADHD is typically reported less in highly religious communities [3], studies have shown that the prevalence of ADHD in the ultra-orthodox Jewish (UOJ) community is similar to that of the general population [4].

The UOJ community is an internally cohesive religious group composed of three major clusters, Hasidim , Lithuanians, and Sephardim and each is further splintered into numerous subgroups. All groups are characterized by a strong adherence to rabbinical authority and cultural codes, which heavily shape their values, beliefs, and behaviors. Study of the *Torah* (religious texts) is believed in the UOJ community as a central value. The men spend a large portion of their time studying religious texts, while women are generally the main breadwinner of the home. This community is growing both in Israel and globally. Currently the UOJ community makes up approximately 10% of Israel’s population, and there are estimates that they will comprise around 30% of Israel's total population within the next three decades [5, 6]. The UOJ community tends to be culturally conservative, maintaining a distinct separation from outside influences. Consequently, they often avoid utilizing public mental health services due to concerns about stigma and a lack of culturally sensitive treatments [7]. This may put them at risk for negative health outcomes and a diminished quality of life (QoL) [8, 9].

The behavior manifestations associated with ADHD are known to negatively affect members of the family unit [10]. Mothers often assume the primary caregiving role for their children and bear a significant portion of the responsibility when raising their child with ADHD [11]. Mothers of children with ADHD experience various burdens, including physical [12], psychological [10], social [13], and functional [2] challenges, which have been shown to adversely affect their psychological health [10] and QoL [14]. The burden of mothering a child with ADHD in the UOJ community has similarly shown to negatively impact these mothers’ psychological health and QoL while additionally facing unique socio-cultural factors influenced by their environment, for example issues related to gender roles, behavior expectations, and culture-related stigma [8, 9]. Studies have emphasized the need for developing culturally appropriate treatments and interventions for this growing and under‑served population, emphasizing the importance of cultural competency. Cultural competency is a contextual and dynamic process where health professionals adapt their practices to address the specific cultural sensitivities and needs of clients through understanding and effective communication. It encompasses cultural awareness, knowledge, and skills [15].

A public health intervention is defined as planned actions to prevent or reduce a particular health problem, or the determinants of the problem, in a defined population [16]. Intervention Mapping is a protocol that guides the development of multi-level health promotion interventions and implementation strategies. It is comprised of six steps which include: (1) performing a needs assessment by identifying what needs to change and for which population; (2) specifying the intervention’s objectives and to which determinants they will address; (3) selecting theory-based intervention methods that match the determinants, and translate them into practical strategies; (4) integrating the strategies into practical material to deliver intervention content; (5) planning for implementation and sustainability of the intervention by identifying key stakeholders and determining what their needs are and how these should be fulfilled; and (6) creating an evaluation plan to measure the intervention’s effectiveness [17]. Intervention Mapping provides an organized and structured approach to intervention development. It helps the final intervention be grounded in theory and helps create content that drives change in targeted behaviors. Furthermore, it includes co-design methods which involves collaborative efforts among researchers, interdisciplinary practitioners, the target population, and policymakers to create a program that addresses the specific needs of the intended population. This often involves conducting focus groups [18, 19]. Consequently, as a response to the negative effects on psychological health and QoL faced by UOJ mothers of children with ADHD, as well as the need for culturally sensitive interventions for this population, a psychological health promotion intervention was created through the utilization of the Intervention Mapping protocol. Therefore, the aim of this study is to outline the development of a theory-driven and culturally appropriate psychological health promotion intervention for UOJ mothers of children with ADHD by employing the Intervention Mapping protocol.

## Methods

### Design

This study employed a mixed-methods design, involving qualitative content analyses of focus groups with stakeholders who shared at least one commonality in each group [20]. Descriptive statistical methods, including the content validity index (CVI) which was used to measure feedback regarding the developed intervention protocol’s relevance, effectiveness, and appropriateness to its objectives and population. The study was approved by the Hebrew University Institutional Review Board (#15112022). Design and reporting of this study were according to the Template for Intervention Description and Replication (TIDieR) checklist [21] and the GUIDED checklist for reporting intervention development studies in health research [22] [Supplementary files 1 & 2].

### Participants

Four homogeneous focus groups consisting of UOJ mothers of children with ADHD, UOJ fathers of children with ADHD, occupational therapists, and educators, participated in this study. Inclusion criteria for (a) mothers and (b) fathers were having a child diagnosed with ADHD aged 6 to 18 and self-identifying as UOJ; (c) occupational therapists provided therapy services to children with ADHD and their families in the UOJ community; and (d) educators had experience working with children diagnosed with ADHD in the UOJ community. Exclusion criteria for mothers and fathers included having a major health condition (aside from ADHD) impacting daily functioning, determined by self-report. Recruitment began after obtaining IRB approval. The study details were advertised on social media within the UOJ community, in UOJ child development centers with administrators' permission, and through the snowball method. Advertisements included study explanations, both authors' credentials, and the first author's contact information. The first author is a PhD student and an occupational therapist who is a member of the UOJ community, and the second author is a researcher in ADHD and QoL. An initial phone call with each respondent ensured participant’s suitability for the study. Informed consent was obtained from all subjects before the study commenced. Focus groups occurred from December 2022 to February 2023.

### Procedure

#### Step 1: conduct a needs assessment

The intervention protocol's development began with a needs assessment, informed by two initial studies on UOJ mothers raising children with ADHD [8, 9], considering the absence of prior research on this topic. These studies included a review of the literature on mothering a child with ADHD in the general population and culturally specific information on the UOJ community. Focus groups were conducted to identify ways to promote psychological health for these mothers and explore potential barriers and enablers to program participation. Prepared in accordance with Krueger & Casey's recommendations [20], a question guide featuring four questions used for all four groups, facilitated discussions. Participants were briefed for five minutes on the research findings by Budman et al. [8] and Budman & Maeir [9] through a two-slide PowerPoint presentation. The objectives were to explore participants’ opinions on previous findings, providing an opportunity to contest them, and to shift the discussion towards what type of intervention would be appropriate for these mothers. An additional objective was to identify potential barriers and facilitators for mothers' health and participation in an intervention program. Participants were asked: (1) What do you think about the research findings? (2) Are there other topics or issues that were not mentioned in the research findings? (3) What can be done to promote the health of these mothers? (4) What are the expected barriers and enablers to the mothers' involvement in an intervention to promote their health?

#### Focus groups process

Before the focus groups commenced, consent forms were emailed to all participants (*n*=25), and signed consent was obtained. Participants then completed a demographic questionnaire. Once all questionnaires were received, suitable times for the focus groups, conducted via Zoom, lasting 80-90 minutes each, were scheduled. Sample size determination, including number of focus groups and participants, was based on relevant intervention development literature [23] and established guidelines for focus group methodology [24]. Sample size adhered to the acceptable range of at least three focus groups with 4-12 participants per group. Multiple heterogeneous groups, involving diverse stakeholders, were chosen to support varied perspectives and data saturation. Transcripts were reviewed and reflected on by both researchers between focus groups to identify any new concepts and support a further comprehensive exploration of concepts in the following focus groups [24]. The first focus group was with occupational therapists, then mothers, father, and lastly with educators. The first author recorded and transcribed all the focus group sessions.

#### Step 2: develop intervention objectives

Deductive content analysis using the combined Behavior Change Wheel’s COM-B system [25] and the Theoretical Domains Framework (TDF) [26] was performed on the focus groups’ qualitative data. The Behavior Change Wheel is a theory that integrates 19 theories of behavior. It’s COM-B system is a framework that categorizes factors that influence ‘behavior’ in terms of ‘capability’ (physical and psychological), ‘opportunity’ (social and physical), and ‘motivation’ (autonomic and reflexive), and postulates that behavior change requires a change in one or more of these three areas. The TDF is a comprehensive framework combining multiple behavior change theories to identify the obstacles and facilitators for implementing behavior change interventions. It is comprised of 14 domains which have been validated as subcomponents of the COM-B system. The combined COM-B system with the TDF subcomponents have been employed in intervention design as predefined codes in qualitative analysis, to identify determinants that need modification to achieve behavior change [23]. Participants’ statements were attributed to the most relevant behavior change domain (deductive content analyses). Coding was performed by both authors. Discrepancies were discussed until final code attribution was agreed on by both authors.

#### Step 3: develop theoretical methods and practical strategies

A matrix, guided by Michie et al.'s [27] intervention design framework, was created to outline theory-based behavioral determinants, intervention objectives and target behaviors. The combined COM-B system and the TDF, carried forward from the previous step, guided the selection of relevant theories and subsequent appropriate Behavior Change Techniques (BCTs) for the intervention. BCTs, active components influencing target behavior change, were chosen from the Behavior Change Techniques Taxonomy v1 (BCTTv1), a taxonomy comprising 93 specific BCTs organized into 16 groups [28]. After specifying target behaviors from behavior domains, each author independently selected BCTs related to the target behavior(s) and theories. Discrepancies were resolved through discussion until consensus was reached on the final set of BCTs.

#### Step 4: intervention production

Step three findings guided the production of the intervention (i.e. activities and materials) by both authors together, and in line with the theories and the set of BCTs. The initial protocol draft, along with a table of specifications ([29]; Supplementary 3), was sent to mothers from the original focus group via email. In the table of specifications, mothers rated each intervention activity for, (a) relevance to session’s objectives, (b) effectiveness in modifying the target behavior, and (c) appropriateness (language, nature of activities, instructions, response choices, etc. are clear and easy to understand) for UOJ mothers of children with ADHD. Items were rated on a four-point Likert scale (1-low to 4- very high), with a section for additional comments. Quantitative data aided in establishing preliminary content validity, measuring the intervention's component activities' relevance and likely effectiveness in achieving objectives for the target population [29].

### Statistical analysis

After completion and return of the table of specifications, statistical analysis, using the content validity index (CVI) and excel, was carried out on the quantitative data. The CVI expresses the proportion of agreement on each item, which is between zero and one. If the total CVI is higher than .79 , the item is deemed appropriate, between .70 and .79, needs revision and if less than .70, is eliminated [30]. Qualitative content analysis was conducted by the two authors for analyzing written recommendations [31] and subsequently a revised version of the intervention protocol was created.

#### Step 5: develop intervention implementation plan

Preliminary steps in developing the implementation plan were carried out in conjunction with step four since planning for implementation is closely tied to the content, target audience, and setting of the program [17]. As this step includes more broad aspects for instance, strategies to support the practicability, appropriateness, resources, equity in access, and sustainability, only an initial implementation plan was developed. The findings from previous studies, the insights gathered from the focus groups, and feedback from the mothers, played a role in partially guiding this phase of development, particularly focusing on the intervention setting.

#### Step 6: create an evaluation plan to measure the intervention’s effectiveness

The evaluation phase plays an important role in assessing the achievement of intervention objectives and requires thorough planning. Analysis and decisions made during the intervention development process are utilized to shape the evaluation plan. By considering the behavioral determinants, objectives, and behavior change theories, suitable measures are chosen to assess the outcomes and the anticipated paths of change. Step six was not included in the current study and will be addressed in a follow-up study.

## Results

### Step 1: Conduct a needs assessment

Two preliminary studies revealed the risk of UOJ mothers of children with ADHD for negative psychological health and QoL outcomes. Budman et al. [8] discovered that over half of the mothers in their study exhibited symptoms of poor psychological health and moderate levels of parental stress. Moreover, 17-37% reported a lack of engagement in activities shown to promote health and QoL, such as personal healthcare, social, productive and leisure activities. These mothers also expressed dissatisfaction with the frequency of their engagement in these activities. Budman & Maeir [9] found that UOJ mothers of children with ADHD faced child ADHD-related challenges which were highly influenced by cultural factors. For example, they described the burden they experienced from their child’s ADHD behavioral manifestations in a highly religious context, that values learning and obedience. They also highlighted social factors including the stigma surrounding their child’s diagnosis, the absence of culturally appropriate interventions, and their role as breadwinner. Motherhood within this community is viewed as a pathway to religious self-fulfillment, and thus, perceived failures in this role may carry additional religious interpretations for these mothers [9, 32]. In addition, they described their beliefs and barriers to their own self-care. In the current study focus groups were conducted to explore how to address the specific needs of these mothers, aiming to promote their psychological health.

#### Summary of focus group participants

Twenty-five participants participated in four focus groups. A description of the participants are outlined in Tables 1 and 2. Each focus group session consisted of homogeneous representation including six–seven participants per group.

**Table 1:** Demographics of focus group participants, mothers

Participant	Stakeholder type	Age	Marriage status	Ultra-orthodox affiliation	Education	Work	Income status	# children	Child medication	Self ADHD
C	Mother	31	married	Lithuanian	BA	Full-time	Average	4	Everyday	Yes
F	Mother	32	married	Hasid	BA	Full-time	Above average	6	Everyday	No
R	Mother	32	married	Lithuanian	Certificate Program	Full-time	Average	5	Everyday	Maybe
Ru	Mother	39	married	Sefardic	BA	No	Average	8	Sometimes	No
Ra	Mother	48	married	Lithuanian	BA	Part-time	Below average	8	No	Maybe
S	Mother	39	married	Lithuanian	BA	Full-time	Average	7	Everyday	Yes

*BA* Bachelor’s degree; National average monthly income per household of seven individuals in Israel is reported at 18,667 nis (Central Bureau of Statistics, 2022); *ADHD* Attention Deficit Hyperactivity Disorder

**Table 2:** Description of focus groups’ stakeholders’ job titles and descriptions, and relationship with ADHD

Stakeholder	Stakeholder type	Sex	Years experience	Work setting	ADHD in family	ADHD self
YZ	Father	M	-	-	Child	No
IR	Father	M	-	-	Child, Wife	No
YC	Father	M	-	-	Child	No
E	Father	M	-	-	Child	Maybe
YO	Father	M	-	-	Child	No
Yi	Father	M	-	-	Child	Yes
E	OT	F	24	Child development clinic Private practice	Child	No
H	OT	F	7	Child development clinic Special education preschool	No	No
Ho	OT	F	3	Child development clinic	No	No
Ti	OT	F	10	Private practice Primary School Supervisor	Brother	No
Ra	OT	F	9	Child development clinic Primary school	No	No
Ri	OT	F	9	Private practice Primary school	-	-
Tl	OT	F	5	Mental health clinic for children and adolescents	Sibling	Yes
A	Teacher	M	16	Primary school High school	Child	No
Sh	Rabbi Principal	M	12	Boy’s primary school	Child	Maybe
C	Educational supervisor	M	12	Boy’s primary school	Child	No
Yf	Teacher	F	14	Girl’s high school	Child	No
Yl	Educational supervisor	F	10	Preschool and kindergarten	Child, Sibling	No
Sa	Principal	F	17	Girl’s primary school	None	No

*ADHD* Attention Deficit Hyperactivity Disorder, *OT* Occupational therapist

### Step 2: develop intervention objectives

Qualitative content analyses of the focus groups transcripts revealed determinants of health promotion behavior change attributed to 10 of the 14 theoretical domains. Most participants made statements relating to the domains of Social Influences (*n*=21), Environmental Context and Resources (*n*=20), Knowledge (*n*=17), Skills (*n*=12), and Social/Professional Role & Identity (*n*=12). See Table 3 for summary examples of statements and related domains.

**Table 3:** Summary of sample statements from focus groups assigned to the combined COM-B and Theoretical Domains Framework

COM-B component	TDF Domain	Sample statements	Focus group	Number of participants
Capability	Knowledge	“Knowledge is a really significant power, probably in everything, but in this situation with my child I feel it very much.” (M-F) “The ambiguity of the diagnosis. Sometimes parents don't know exactly how it affects their day-to-day life, providing this information is very reassuring… there is a need for psycho-education”(O-H) “It is important to learn what research says, and what treatment options there are.”(F-YC) “Often there is a lack of coherence about when the child succeeds or when not…there is a gap between what they think their child can do and what the child is realistically capable of”(O-E) “Sometimes parents are told ADHD, but they hear the letters of the English alphabet, A.B.C. They need it to be explained and how their child can be helped.” (E-Yl)	Mothers	6
			Occupational therapists	5
			Fathers	3
			Educators	3
	Skills	“I need to have tools to use for my child” (M-F) “Mothers need tools, to know what to do” (O-E) “They need to advocate and know how to navigate the educational system”(O-H) “The mothers need a lot more skills until it (the medication) starts working. In the morning getting ready, and when he comes back from the school when his medication has worn off.” (F-YZ)	Mothers	4
			Occupational therapists	4
			Fathers	4
	Memory, Attention, and Decision Processes	“Things started to unravel, working in the afternoon at home with four little ones, I collapsed, and some good people took me to a psychiatrist, because they thought I had postpartum depression. But I left the psychiatrist with a prescription for Ritalin. He told me, You don't have depression. You have severe attention deficit disorder”(M-C)	Mother	1
			Occupational therapist	1
			Father	1
			Educators	3
	Behavioral Regulation	“Often times the mother has ADHD. A mother comes for her child, but the conversation is surrounding *her experiences and pains and not the child*…She is so overwhelmed, it’s harder to work with these mothers.” (E-Yf)	Educator	1
Opportunity	Social Influences	“Like in many areas of the ultra-orthodox community, there is no ability to talk about it (ADHD) in a way that seems normative - to normalize the phenomenon”(M-R) “Often times parents in the ultra-orthodox community don't want other people to know their child has ADHD…there is a lot of hiding and that makes things even more difficult.”(O-Ti) “There is no place for these children’s differences. There is no place to be different but they are different. And the mother feels that she is different.”(M-Ru) “There are cultural codes in the community and so a child who doesn’t follow the codes leads the mother to say, ‘My child is a problem’, and they are unable to see their child holistically” (O-Tl) “It is very possible that there is more than one child with ADHD. There is a lot of stress for parents” (O-Ri) “Since we know it's (ADHD) hereditary, parents often come with their own with difficulties. So if the dad is having difficulties, he is less of a partner to help, she has to deal with both him and the children.”(O-Ti) “It falls mainly on the mother, taking the child to treatments, and receiving parental guidance. I don’t usually see the fathers… the mother is the one at home and sees the child.” (O-Ra) “I think the mother is the foundation who supports the child's regulation. And sometimes I feel that they don't really feel that it's legitimate (in their environment) for them to say: Wait, I'm here too, and what about me?”(O-E) “Society says it's your fault that your daughter behaves like this, your daughter is without limits, so you’re a bad mother.”(M-Ra) "For many boys the expectation is that he must continue to learn for the next twenty years, to sit in a room and study.”(O-Tl) “They (educational institution) have no patience and they always advocate for medication. They told us, ‘Listen, there is a pill, it's called Ritalin, it works.” (F-Yi) “The biggest problem is that the high schools that weaker boys end up going to, are for troubled boys. Part of the urgency of doing well academically (via medication) is that they will get into a yeshiva that will be a better environment. It is not just about the learning; it’s about getting into that safer environment.” (E-Sh)	Mothers	5
			Occupational therapists	6
			Fathers	5
			Educators	5
	Environ-mental Context and Resources	“Many parents do not know who to go to. They don't feel like they're really being listened to. They (healthcare system) throw them the prescription.”(M-S) “Looking back, a community of parents for me would have provided an excellent and supportive answer, it would have directed me and provided a short-cut to getting help.”(M-Ra) “I must have time for myself to go out alone, go out with my husband, buy myself clothes, put on makeup, feel good about myself, have time for myself, and not always just around this whole topic.”(M-F) “As much as I tried to talk to the (educational) staff, they don't have the tools, they're not even to blame, but they just don't have the tools to deal with our children”(M-Ru) “There is pressure around the issue of boy’s learning Torah, compared to public schools where there are many sports and all kinds of classes beyond studying Torah all day.”(O-Ra) “They (educational institution) have more than 25 children in the class, and they don’t have patience for it (ADHD). They study ten hours straight, so take something (medication). I'm just saying it's the reality.”(F-IR) “If a child has untreated ADHD, it requires something from the educational system that it doesn’t always have. Sometimes the child will go to a special educational class because the child cannot sit for a minute, the parents need to understand that.” (E-Yl) “A child who is a little different gets in the way, he's frustrated, he can't learn, the parent is frustrated and it's a never-ending cycle. With us (UO), it is very square and there is not much to do. If parents understand this, and follow the educators’ advice, it will save the child. If not, then the child will leave the fold, kind of dropping out the world of the Torah.” (E-Sh) “Full cooperation with parents, is what I think will reduce the gap significantly.” (E-Sa) “It's not always appropriate (for the mothers) to talk to the Rabbis in the school. Communication is through the father. It’s like broken telephone.” (O-Tl) “The father is needed for the studies, the mother doesn't really have a grasp of where the children (boys) are with the study materials, so naturally it’s the father that really has to be more involved.” (F-Yi) “Since fathers don’t work, they come to the therapy session. It’s hard to find a time that the mother could come to a session, because she works, and the father does not work.”(O-H)	Mothers	6
			Occupational therapists	5
			Fathers	4
			Educators	5
Motivation	Social/Professional Role & Identity	“Because a parent thinks, I must take care of the child, this is my life's mission” (M-S) “I feel that all day I am having to manage my daughter, be a manager, in addition to my other roles, I have a job as a manager for my child.”(M-Ra) “To have the strength to go to a speech therapist (for child), I need the strength for myself. ‘Where am I in all of this’?” (M-S) “Her role includes talking to doctors most of the time and takes him to school. At home she deals with his behavior when he comes back from school. Whereas the father is more involved in his education.” (F-E) “The mothers’ role is really the central one. Mothers send their children off in the morning and receive them after school. If she sends her child off to school medicated or if she realizes that this child really needs help, then everything can change. It’s precisely the mothers who are much more sensitive, have much more emotion and understanding.” (E-A)	Mothers	4
			Occupational therapists	2
			Fathers	4
			Educators	2
	Beliefs about Capabilities	“Only my child is not successful. What does this mean about me as a mother?’”(O-Tl)	Mothers	5
			Occupational therapists	2
	Goals	“It is important to get to the mothers’ health through their children. Emphasize the child. Mothers may not go to a group to get help, even once a week, but if it's for their child… then it’s a gateway to her, her needs, without her "noticing.”(O-H)	Occupational therapists	2
	Emotion	“If my child is not well, then I am not well. Because for us, it’s our mental health, because we are mothers, so we want our children to be well.” (M-C) “Our sense of failure as parents…I feel damaged that I'm not a mother with boundaries”(M-S) “It (mothers’ experience) is something emotional and deep that requires in-depth care and understanding, it's complex. If the mother is emotionally drained, she cannot support her child.” (E-Yf)	Mothers	3
			Educators	2

‘M’ indicates sample quote by a mother; ‘O’ indicates sample quote by an occupational therapist; ‘F’ indicates sample quote by a father; ‘E’ indicates sample quote by an educator

Based on the outcomes of step one, the intervention's overall goal was to promote the psychological health of UOJ mothers of children with ADHD. The objectives were developed taking into consideration the frequency of determinants that were expressed by participants. Specifically, challenges related to social influences such as misattributions of child ADHD behaviors to mothering abilities and knowledge about ADHD were frequently expressed. Hence, key objectives were to address and reduce stigma surrounding ADHD and increase knowledge about the biological basis of ADHD. Additionally, many participants, including parents and therapists, shared frustrations regarding child ADHD-related unmet needs in the schools. While educators acknowledged resource constraints, they also recognized the need for collaborative mother-school partnerships. However, mothers’ limited access to teachers were also identified as a barrier in communication and partnership with the staff. Thus, an additional set of objectives addressed these contextual issues such as enhancing awareness to the school systems’ capabilities, resources and limitations, identifying key educational staff available to mothers, and enhancing mothers’ advocacy skills. Lastly, mothers' self-care challenges were highlighted. To address this, an objective was added to foster mothers' involvement in self-care activities. The intervention’s objectives and determinants they address are listed in Table 4.

**Table 4:** Intervention objectives and related determinants according to the Theoretical Domains Framework

Objective(s)	Determinant(s), TDF
Reduce shame/stigma	Social influence, Emotion
Modify negative beliefs about mothering and its relationship to child participation	Social influences, Emotion
Promote positive identity regarding their mothering role	Social/Professional Role & Identity
Reduce ambiguity of diagnosis and behavioral manifestations	Knowledge
Identify reliable resources regarding optimal and recommended treatment for child’s ADHD	Knowledge, Skills, Environmental Context and Resources
Identify resources available to support mothering a child with ADHD	Knowledge, Environmental Context and Resources
Recognize the gap between the school system’s capabilities and their child’s needs	Knowledge, Environmental Context and Resources
Increase mother advocacy skills	Skills
Promote engagement in self-care activities	Emotion, Knowledge, Skills, Environmental Context and Resources

*TDF* Theoretical Domains Framework [26]; *ADHD* Attention Deficit Hyperactivity Disorder

Once the intervention objectives and related list of determinants were generated, a matrix was constructed. Target behaviors that needed to be changed or learned to achieve the objectives were selected following the Behavior Change Wheel framework and guide by Michie [27]. An example item of the matrix can be seen in Table 5.

**Table 5:** Matrix of the learned and behavioral change objectives for ultra-orthodox Jewish mothers of children with ADHD, example item

Problem	Objective	Combined COM-B & TDF	Target behavior	Who performs behavior	What needs to change	When do they perform behavior	Where do they perform behavior	With whom do they perform behavior
Insufficient knowledge of the ADHD condition	Reduce ambiguity of ADHD diagnosis and behavioral manifestations	Capability: Knowledge	Members will use biological attribution and research-based descriptions/language when discussing their child’s ADHD manifestations	Members of the group	1) Gain research-based knowledge about ADHD condition 2) Use of appropriate language and labelling to attribute child’s challenges to ADHD	When speaking about ADHD regarding their child specifically	In group and out of group by members’ self report	Self and with others including members of the group, members’ spouses and educational staff

*ADHD* Attention Deficit Hyperactivity Disorder; *COM-B* The Behavior Wheel’s system, Capabilities, Opportunity, Motivation, Behavior (Michie et al., 2011); *TDF* Theoretical Domains Framework (Cane et al., 2012)

### Step 3: develop theoretical methods and practical strategies

**Fig. 1:**
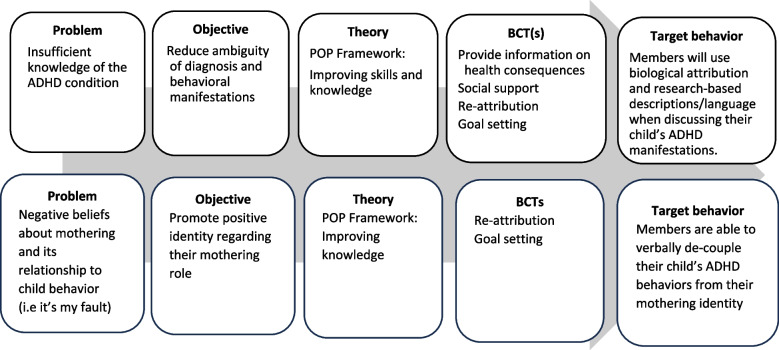
Behavior Change Techniques and their relationship to the intervention’s objective, theory, and target behavior, examples. Note. POP framework: Parenting Occupations and Purposes framework [34]

After developing the matrix, the authors identified theories that support implementation of intervention objectives as well as provide strategies to achieve them. Two theories were selected that focus on social learning and the parenting role, the Social Learning Theory [33] and the Parenting Occupations and Purposes (POP) framework [34]. Social Learning Theory describes a dynamic interplay between personal factors (thoughts, beliefs, and emotions), social factors, and behavior. The theory emphasizes the centrality of self-efficacy as a behavior change agent. Sources of self-efficacy include vicarious experience, social persuasion, performance accomplishments, and physiological and emotional states. Therefore, a peer group setting was chosen as a behavior change strategy to provide the opportunity to learn from the outcomes and experiences of others, receive feedback, fosters beliefs in predicting success, and motivate intentional behavior change [33]. The POP framework describes parenting occupations that directly and indirectly address the child’s basic, developmental, and social needs. Continuous Parental Development (CPD) is required to enhance parental capacity and includes seeking assistance, managing resources, improving skills and knowledge, and practicing self-care [34]. Strategies in the intervention were aimed to mainly support CPD and included (1) gaining knowledge about ADHD, (2) identifying support resources, (3) developing communication skills, and (4) promoting personal self-care to enhance the ADHD mothering role. To inform the selection of strategies, the authors referred to the Behavior Change Techniques (BCTs) identified by Michie et al. [28]. They selected specific BCTs to address the intervention’s objectives such as providing information on health consequences, social support, re-attribution, active learning, goal setting, and promoting an identity associated with the desired behavior change. The authors then developed practical implementation strategies, consisting of specific activities aligned with the selected BCTs. An example in Fig. 1 illustrates how the BCTs employed by the intervention contribute to achieving the related behavior change objectives, are related to theory, and target the behavior.

### Step 4: intervention production

The choice of a psychoeducation group intervention stemmed from participants statements of mothers’ needs and is in line with the theories in step three. The intervention was structured around the information from the previous steps along with a logical sequence to effectively address the objectives and achieve target behaviors. Subsequently three modules were developed for the intervention. The first module is “Developing knowledge of ADHD and it’s biological origins”. Content in this module focuses on: (a) mothers’ reduction of shame and stigma surrounding their child’s ADHD condition, (b) gaining knowledge regarding the ADHD condition and optimal treatment recommendations, (c) identifying support resources for ADHD mothering skills, (d) and promote a positive identity regarding their mothering role. This was selected as the first module to build mothers’ interest and motivation by focusing on determinants that were most stated in the focus groups as effecting mothers’ psychological health (see Table 3) and to provide a base of knowledge for the subsequent modules. The second module is “Developing strategies to manage child ADHD in the school”. Content in this module focuses on mothers’ identifying and communicating with key persons in their child’s school along with learning advocacy skills [35]. This module targeted determinants related to Skills, Environmental context and resources, and Social/Professional Role & Identity. The third module is “Healthy mother-healthy child: Legitimacy and importance of self-care.” Content for this module focuses on promoting mothers’ engagement in health promoting activities in response to their caregiver role and is in line with the POP framework [34]. It targets the determinants of Emotion, Knowledge, and Environmental context and resources. The full program implementation spans six weeks, with module one lasting four consecutive weeks, followed by one week for module two and one week for module three. The program is led by an occupational therapist trained in delivering the content and group therapy techniques. Each session is frontal and two-hours in length which includes a short presentation, group activities, reflection, and goal setting. Educational materials consist of binders containing session presentations and client diaries for personal notes and goals. Each group consists of seven-eight participants, adhering to recommended guidelines for group therapy [36].

All the mothers provided feedback which confirmed the overall relevance (CVI^avg^= .86), effectiveness (CVI^avg^= .85), and appropriateness (CVI^avg^= .87), of the activities in all three modules. Descriptive feedback provided additional insights. Mothers overall stated positive feedback on the intervention protocol, for example the number of sessions and length of each session were stated as appropriate for mothers’ busy schedules. However, mothers also provided some suggestions for consideration, for example mothers’ own ADHD profile was heighted as a potential barrier for goal(s) attainment. As such an adaptation was made and goal(s) will be related to in the sessions only and not as additional tasks to be completed outside the sessions. Furthermore, there were varying statements reflecting a tension between whether the group should serve as also a support group or only as a source of professional information. Additionally, some mothers expressed concern that it shouldn't replace the role of professional therapy and medical treatment and as such should be clearly stated. Consequently, in advertising and at the beginning of the intervention's first session, a clear statement was included, affirming that the group serves as both a social support and a source of professional information, without replacing other therapy and medical treatment. Mothers also highlighted the significance of inviting the appropriate educational staff member to the group, someone who holds a key role and is willing to actively listen and provide assistance. This was stressed due to the inconsistency of having such an individual in every school setting. The Intervention outline can be seen in Table 6.

**Table 6:** Intervention protocol content outline

Unit	Session	Objective(s)	Target behavior	Method
Developing awareness/knowledge of ADHD and it’s biological origins	1	Create a safe emotional environment to reduce shame/stigma	Member shares thoughts and experiences to the group.	1) **Setting**: safe and attractive place in the school building 2) **Group contract:** a) Therapist brings prepared contract which includes establishing boundaries, confidentiality, communication, norms of behavior, expectations from the group, overarching goals. b) Members modify and agree through group discussion. 3) **Activity:** Members complete the ADHD stigma questionnaire and then have a follow up discussion 4) **Introduction to client diary:** A place for each member to write down their takeaways and mini goals during each session
	2	Reduce ambiguity of diagnosis and behavioral manifestations	Members will use biological attribution and research-based descriptions/language when discussing their child’s ADHD manifestations.	1) **Short presentation:** provide background information on ADHD 2) **Group Activity:** a) Analyze prepared ADHD script of behavioral manifestations b) Members write a script together describing ADHD along with examples of ADHD behavioral manifestations (from personal experiences). 3) **Goal:** Have a real or imagined conversation with a person of their choice and describe their child’s ADHD utilizing labelling/language from this session
	3	Identify reliable resources regarding optimal and recommended treatment for child’s ADHD Identify resources available to support mothering a child with ADHD	Members identify 1-2 appropriate resources for their child’s ADHD challenges and/or parenting skills.	**1) Short presentation:** a) Recommended ADHD treatment, clinical guidelines, examples of behavioral ADHD treatment options b) Guest participant: Guidance counselor (or appropriate school staff) to explain service (content and process) 2) **Reflective group activity:** a) Identify reliable sources of ADHD and health information b) Recall successful past experiences finding reliable sources 3) **Establish the group as a resource:** a) Follow up norms of communication with the group during and post intervention 4) **Goal:** each member creates an action plan to identify 1 resource for managing their child’s ADHD.
	4	Modify negative beliefs about mothering and its relationship to child behavior Promote positive identity regarding their mothering role	Members are able to verbally de-couple their child’s ADHD behaviors from their mothering identity	1) **Group Activity:** a) Members are presented with statements/depictions that reflect the cognitive bias (i.e., mothers are to blame for child’s behavior) in various social contexts. b) Members generate personal examples of scenarios c) Members create alternate scenarios- role play positive reframing of belief in response to child’s behavioral challenges (e.g. child is not responding to parenting attempts at routinizing bedtime as opposed to self-blame, ‘I’m not a good enough mother to this child’- mother recognizes need for additional/knowledge/skills) 2) **Goal:** each member identifies either an example from a personal scenario or from the media displaying cognitive bias moving from self-blame to inquisitive stance
Developing strategies to manage child ADHD in the school	5	Understand the gap between the school’s capabilities to meet their child’s needs Increase mother advocacy skills	Members will identify a person of interest in their child’s education and be able to advocate for 1 of their child’s needs.	1) **Short presentation given along with key school staff member:** Brief education about effective self-advocacy 2) **Role playing:** a) Members identify a need of their child with ADHD b) Practice the skill of self-advocacy (multiple scenarios, different contexts) 3) **Goal:** Each member identifies in own life: a) Where they want to apply this b) Create a plan
Healthy mother-healthy child: Legitimacy and importance of self-care	6	Promote engagement in health promoting activities in response to their caregiver role	Members will increase engagement in 1 health promoting activity (subjectively or objectively)	1) **Short presentation:** a) The parenting occupations and purposes framework b) Knowledge on link between healthy lifestyle behaviors and health outcomes 2) **Group activity:** Adapted Occupational Questionnaire to build awareness of personal profile regarding engagement in health promoting activities and discussion 3) **Goal:** a) Each member chooses a goal* to promote their engagement in a health promoting activity b) Action plan: setting + visualize implementation, identify barriers, identify resources *Therapist provides a bank of goals to choose from.

The parenting occupations and purposes framework (Lim et al., 2021)

### Step 5: develop intervention implementation plan

This step was only partially completed and focused mainly on the intervention setting and guest participant. The intervention setting will be within the ADHD child's school. This is to help foster closer partnership between mothers and school staff, aligning with the shared responsibility of supporting the ADHD child. This approach was supported by both mothers and educators in the focus groups. Consumers will be mothers of children with ADHD from the same school (primary or high school), as they likely share similarities in experiences related to religious and educational contexts. Groups are most effective when participants present with similarities and can be considered peers [36]. In addition, as suggested by mothers, a school staff member, either the principal or guidance counselor, will participant (see Table 6) in modules one and two to facilitate communication between mothers and school staff [36].

### Step 6: create an evaluation plan to measure the intervention’s effectiveness

This step was not included in the current study. To evaluate the feasibility and effectiveness of the intervention, a pilot study with a pre-test post-test design will be conducted in a follow-up study. As 20-25 participants is recommended in the literature regarding pilot studies researching intervention efficacy [37], three groups of seven to eight UOJ mothers of children with ADHD will be included. By considering the overall goal of promoting mothers’ psychological health, objectives, behavioral determinants, and behavior change theories, suitable measures will be chosen to assess the outcomes, the anticipated paths of change, and validity and reliability of the intervention.

## Discussion

Intervention Mapping was used to create a psychological health promotion intervention tailored to UOJ mothers of children with ADHD. This was accomplished through its comprehensive and systematic process that provided a framework to address the multifaceted aspects related to mothering a child with ADHD in the UOJ community. Steps one through four were described in the current study along with preliminary and limited aspects from step five. Step six was not included and will be addressed in a further up study. Utilizing this process allows for the incorporation of co-design methods, and health promotion initiatives created with input from the target population and key stakeholders tend to have higher chances of successful implementation and effectiveness [18]. Consequently, this comprehensive process highlighted various determinants that impact these mothers, such as insufficient knowledge about ADHD, limited skills in identifying appropriate treatment and resources, challenges in communication with educators, stigma, and barriers to maternal self-care. As a result, these determinants shaped the intervention's objectives and aided in the identification of strategies to help modify behaviors and address barriers, while considering their distinct cultural context.

One of the intervention’s main objectives was to reduce ambiguity of the ADHD diagnosis and its behavioral manifestations. Psychoeducation programs that enhance knowledge, coping strategies and promote better maternal health have shown success among mothers of children with disabilities [38]. Having information regarding the ADHD condition and its recommended treatment options was specifically emphasized in the focus groups as an important factor affecting the health and QoL of UOJ mothers of children with ADHD. This may be attributed to the cultural conservatism and limited exposure to external influences in the UOJ community, which may restrict access to the internet and available resources due to filters and other limitations [38]. Consequently, parents in this community may encounter difficulties in obtaining comprehensive information about ADHD and accessing recommended treatment guidelines [15]. To address this challenge, it was an important objective to not only provide knowledge but also equip mothers with skills to identify and seek out information and resources within the framework of what is culturally acceptable in their community.

In addition, the intervention was developed in a group format to foster maternal self- efficacy [33, 36] along with creating a peer group to support normalization of the ADHD experience among the mothers. Since there is stigma surrounding ADHD in the UOJ community, mothers report “hiding” their child’s condition [9]. This was also indicated in the focus groups in the current study, however mothers also stated wanting a peer community for support. Thus, it underscored the appropriateness of a group setting where mothers can openly discuss their experiences with a peer group in a normative manner [36, 38]. Nonetheless, mothers' feedback on the intervention indicated that their primary need was acquiring information and skills to effectively manage their child's ADHD, not solely participating in a "support group." This apparent contradiction between seeking a supportive community and not wanting a "support group" might reflect the mothers' lack of legitimization of their personal needs and time allocation. This tension was addressed by the addition of a statement when advertising and at the start of the first session in module1 for clarity and establishing appropriate expectations. UOJ mothers have expressed limited time availability to activities outside of their mothering and working roles, thus their identity as caregivers tends to prioritize child-focused activities over their own needs [9]. As such, while stating they want a supportive community to support their mothering experience, it might be preferrable to emphasize the primary child-focus of the group in order to legitimize participation.

An additional barrier that emerged in all the focus groups was the challenges mothers face in navigating the educational institutions, particularly in terms of the school's capacity to support their ADHD child's needs and the mothers' limited ability to communicate with their child's educators, especially regarding their sons. The UOJ community adheres to specific social norms that emphasize modesty, education, and gender segregation. It is customary for men and women who are not family members to refrain from communicating with each other [32]. Additionally, there is a distinction in the educational curriculum for boys and girls, with boys focusing more on religious texts such as *"Torah and Talmud"* and less on secular subjects. In contrast, girls' education places more of an emphasis on secular studies to equip them with the necessary skills for future professions, enabling them to fulfill the role of breadwinner for their families [39]. These factors often result in fathers taking the lead in engaging with their son's educators. Mothers, however, often have more information regarding their ADHD child’s healthcare and function and as such having decreased communication with their son’s educators may lead to insufficient sharing of important information. In a previous study by Budman & Maeir [9] and in the study’s current focus groups, mothers described unsatisfactory communication with the school as negatively affecting their psychological health and QoL. Therefore, it became clear that it was important to include objectives aimed at increasing awareness of the school system's capacity to meet the needs of children with ADHD and provide mothers with culturally sensitive communication skills for effective engagement with the school. These objectives are in line with the concept of Sense of Coherence, which represents an adaptive dispositional orientation within an individual that supports coping with challenging experiences. It encompasses the individual's perception of the meaningfulness (worthy of investing effort), comprehensibility (structured and predictable), and manageability (have the resources to meet the demand) of a situation. When individuals can comprehend, manage, and find meaning in an experience, they have a higher likelihood of coping successfully [40], and thus support positive health outcomes [41].

The intervention included a module focused on promoting engagement in health-promoting activities to support the self-care and health of these mothers. It is noteworthy that while the significance of mothers' self-care was acknowledged, the intervention primarily emphasized addressing their knowledge and skills related to ADHD and managing their child in the school system. This aligns with the existing literature describing cultural nuances and roles in the UOJ community. The legitimacy of mothers' self-care is often intertwined with their roles as mothers and partners in that they report prioritizing self-care activities as a means of enhancing their ability to care for their family members [9, 32]. While the literature emphasizes the importance of parent-focused interventions, such as self-care, for improving the health and QoL of mothers of children with disabilities [38], the use of Intervention Mapping and co-design methods allowed for the identification and integration of the cultural needs specific to the target population. This can be seen the intervention modules sequence and weight. Module one addresses providing knowledge and skills regarding the child ADHD condition and its treatment options and is made up of four sessions, while mothers’ self-care is addressed in the last module comprising of one session. Thus, the intervention developed was able to respectfully address the target population’s distinct preferences and cultural sensitivities, while trying to minimize researchers bias and beliefs.

## Limitations

While the utilization of intervention mapping proved beneficial, there are several limitations that should be acknowledged. The overall sample size was small and future research should involve a larger number of participants to effectively incorporate more diverse views. Achieving a balanced representation of participants posed challenges due to the diverse sub-groups within the UOJ community [42]. While participants represented the three main clusters of Hasidim, Lithuanians, and Sephardim there are additional subgroups within each, thus it was beyond the scope of this study to incorporate all possible UOJ sub-groups. Consequently, the views, experiences, comments, and recommendations not fully encompassing the entire UOJ community, potentially limit the generalizability of the findings. Another limitation pertained to the fact that feedback on the intervention was obtained only from the mothers. As mothers are the target population and as such their feedback was most relevant, their small group size indicates that the intervention's content validity is only preliminary. Future research on the intervention's validity and reliability should involve a larger number of participants. Furthermore, the scarce scientific literature on the experiences of mothering a child with ADHD in the UOJ community required the study to heavily rely on literature from the general population, two preliminary studies, and the present study. As the intervention was primarily grounded in these sources, further research is advisable to assess its effectiveness, including a comprehensive evaluation using established and validated metrics. Additionally, the complete Intervention Mapping process was not conducted in this study. Developing an implementation plan (step 5) was only partially completed and other aspects including strategies supporting, for example, the practicability, appropriateness, resources, equity in access, and sustainability of the intervention were not presented. The last step of creating an intervention plan (step 6) was also not included and further research should include both these steps.

## Conclusions

Utilization of the Intervention Mapping protocol facilitated the development of a health promotion intervention for UOJ mothers of children with ADHD that aligns with behavioral change theories. This systematic process, which included co-design methods, enabled the identification of needs, establishment of objectives, and development of implementation techniques and content. Importantly, it allowed for the integration of cultural nuances specific to a minority group. Results informed the intervention’s objectives which included reducing the ambiguity of the ADHD condition by increasing knowledge, enhancing skills in treatment and resource identification, improving communication with educators, reducing stigma, and promoting mothers’ engagement in health-promoting activities. The present study demonstrates that Intervention Mapping is a viable tool for developing evidence-based and theory-driven interventions tailored to minority populations. Future steps will involve evaluating the intervention's feasibility, effectiveness, validity and reliability, and disseminating the findings to inform further interventions and practices.

### Supplementary Information

**Supplementary Material 1.** (DOCX 65 KB)

**Supplementary Material 2.** (DOCX 149 KB)

**Supplementary Material 3.** (DOCX 38 KB)

## Data Availability

The datasets used and/or analyzed during the current study are available from the corresponding author on reasonable request.

## Abbreviations

ADHD: Attention Deficit Hyperactivity Disorder
UOJ: Ultra-orthodox Jewish
QoL: Quality of life
CVI: Content validity index
TDF: Theoretical Domains Framework
BCT: Behavior Change Technique
POP: Parenting Occupations and Purposes framework
CPD: Continuous Parental Development
