# Supplementary material for: Development of a psychological health promotion intervention for ultra-orthodox Jewish mothers of children with ADHD using the intervention mapping protocol
Source: BMC Public Health. 2024 Feb 29;24:645. doi: 10.1186/s12889-024-18126-4 (PMC10905867; doi:10.1186/s12889-024-18126-4)
Supplement: Supplementary file 3 — Supplementary Material 3. (DOCX 38 KB) [file 12889_2024_18126_MOESM3_ESM.docx]

**Table of Specifications**

(Adapted from Kassam-Adams et al., 2012)

Thank you for agreeing to serve as an expert reviewer for the health promotion intervention protocol for mothers of children with ADHD in the ultra-orthodox community. We greatly appreciate your contribution to the development of this intervention. Your input on this survey will help us to evaluate the content validity of the intervention – the extent to which intervention materials and activities match our program goals.

**Directions**

Ratings for each intervention activity:

For purposes of this survey, we have identified INTERVENTION ACTIVITIES which are designed to address the INTERVENTION GOAL(S).

Please rate each ACTIVITY on a scale of 1-4 scales based on the following criteria:

**RELEVANCE to the proposed program goals**: extent to which each specific intervention activity is pertinent to the intended INTERVENTION GOALS(S). (Score = 1- irrelevant to the goal, to 4-essential to the goal)

**EFFECTIVENESS of intervention activities**: likelihood that each specific activity will successfully modify the intended INTERVENTION GOAL(S). (Score = 1- not likely to be effective, to 4-very likely to be effective)

**APPROPRIATENESS for ultra-orthodox mothers of children with ADHD**: extent to which language, nature of activities, instructions, response choices, etc. are clear, easy to understand, and appropriate for these mothers. (Score = 1- unsuitable to these mothers, to 4-very suitable to these mothers)

Additional Comments:

There is room in each section for any additional comments -- we welcome feedback or suggestions based upon your expertise. Feedback on the intervention as a whole is also welcome.

**Intervention protocol content**

| **Unit** | **Session** | **Goal(s)** | **Determinant(s)** | **Target behavior** | **Method** | **Content Validity** | | | |
| --- | --- | --- | --- | --- | --- | --- | --- | --- | --- |
| Developing awareness/  knowledge of ADHD and it’s biological origins | 1 | Create a safe emotional environment to reduce shame/stigma | Social influence | Member shares thoughts and experiences to the group. | 1) **Setting**: creating an attractive setting.  a) Coffee and tea  b) Light healthy refreshments  c) Pretty tablecloth  d) Nice smelling fragrance | **Relevance** | | | |
|  |  |  |  |  |  | 1 | 2 | 3 | 4 |
|  |  |  |  |  |  | **Effectiveness** | | | |
|  |  |  |  |  |  | 1 | 2 | 3 | 4 |
|  |  |  |  |  |  | **Appropriateness** | | | |
|  |  |  |  |  |  | 1 | 2 | 3 | 4 |
|  |  |  |  |  | 2) **Group contract:**  a) Therapist brings prepared contract which includes establishing boundaries, confidentiality, communication, norms of behavior, expectations from the group, overarching goals.  b) Members modify and consent through group discussion. | **Relevance** | | | |
|  |  |  |  |  |  | 1 | 2 | 3 | 4 |
|  |  |  |  |  |  | **Effectiveness** | | | |
|  |  |  |  |  |  | 1 | 2 | 3 | 4 |
|  |  |  |  |  |  | **Appropriateness** | | | |
|  |  |  |  |  |  | 1 | 2 | 3 | 4 |
|  |  |  |  |  | 3) **Activity:**  a) Members complete the ADHD stigma questionnaire  b) Members have a follow up discussion | **Relevance** | | | |
|  |  |  |  |  |  | 1 | 2 | 3 | 4 |
|  |  |  |  |  |  | **Effectiveness** | | | |
|  |  |  |  |  |  | 1 | 2 | 3 | 4 |
|  |  |  |  |  |  | **Appropriateness** | | | |
|  |  |  |  |  |  | 1 | 2 | 3 | 4 |
|  |  |  |  |  | 4) **Introduction to client diary:** Members each have a diary that they write down their takeaways and mini goals from each session | **Relevance** | | | |
|  |  |  |  |  |  | 1 | 2 | 3 | 4 |
|  |  |  |  |  |  | **Effectiveness** | | | |
|  |  |  |  |  |  | 1 | 2 | 3 | 4 |
|  |  |  |  |  |  | **Appropriateness** | | | |
|  |  |  |  |  |  | 1 | 2 | 3 | 4 |
|  | Additional comments: | | | | | | | | |
|  | 2 | Reduce ambiguity of diagnosis and behavioral manifestations | Knowledge  Environmental resources | Members will use biological attribution and research-based descriptions/language when discussing their child’s ADHD manifestations. | 1) **Short presentation:** Provide background information on ADHD | **Relevance** | | | |
|  |  |  |  |  |  | 1 | 2 | 3 | 4 |
|  |  |  |  |  |  | **Effectiveness** | | | |
|  |  |  |  |  |  | 1 | 2 | 3 | 4 |
|  |  |  |  |  |  | **Appropriateness** | | | |
|  |  |  |  |  |  | 1 | 2 | 3 | 4 |
|  |  |  |  |  | 2) **Group Activity:**  a) Analyze pre-prepared sample script of ADHD behavioral manifestations  b) Members discuss personal examples of ADHD behavioral manifestations and then chose one example for the group to analyze how the behaviors relate to ADHD. | **Relevance** | | | |
|  |  |  |  |  |  | 1 | 2 | 3 | 4 |
|  |  |  |  |  |  | **Effectiveness** | | | |
|  |  |  |  |  |  | 1 | 2 | 3 | 4 |
|  |  |  |  |  |  | **Appropriateness** | | | |
|  |  |  |  |  |  | 1 | 2 | 3 | 4 |
|  |  |  |  |  | 3) **Mini goal:** Have a real or imagined conversation with a person of their choice and describe their child’s ADHD utilizing labelling/language from this session | **Relevance** | | | |
|  |  |  |  |  |  | 1 | 2 | 3 | 4 |
|  |  |  |  |  |  | **Effectiveness** | | | |
|  |  |  |  |  |  | 1 | 2 | 3 | 4 |
|  |  |  |  |  |  | **Appropriateness** | | | |
|  |  |  |  |  |  | 1 | 2 | 3 | 4 |
|  | Additional comments: | | | | | | | | |
|  | 3 | Identify reliable resources regarding optimal and recommended treatment for child’s ADHD  Identify resources available to support the ADHD mothering skills | Knowledge  Skills  Environmental Resources | Members identify 1-2 appropriate resources for their child’s ADHD challenges and/or parenting skills | **1) Short presentation:**  a) Recommended ADHD treatment, clinical guidelines, examples of behavioral ADHD treatment options  b) Guest speaker: Guidance counselor or healthcare provider to explain service (content and process) | **Relevance** | | | |
|  |  |  |  |  |  | 1 | 2 | 3 | 4 |
|  |  |  |  |  |  | **Effectiveness** | | | |
|  |  |  |  |  |  | 1 | 2 | 3 | 4 |
|  |  |  |  |  |  | **Appropriateness** | | | |
|  |  |  |  |  |  | 1 | 2 | 3 | 4 |
|  |  |  |  |  | 2) **Reflective group activity:**  a) Identify reliable sources of ADHD and health information  b) Recall successful past experiences finding reliable sources | **Relevance** | | | |
|  |  |  |  |  |  | 1 | 2 | 3 | 4 |
|  |  |  |  |  |  | **Effectiveness** | | | |
|  |  |  |  |  |  | 1 | 2 | 3 | 4 |
|  |  |  |  |  |  | **Appropriateness** | | | |
|  |  |  |  |  |  | 1 | 2 | 3 | 4 |
|  |  |  |  |  | 3) **Establish the group as a resource:**  a) Establish the norms of communication with the group during and post intervention | **Relevance** | | | |
|  |  |  |  |  |  | 1 | 2 | 3 | 4 |
|  |  |  |  |  |  | **Effectiveness** | | | |
|  |  |  |  |  |  | 1 | 2 | 3 | 4 |
|  |  |  |  |  |  | **Appropriateness** | | | |
|  |  |  |  |  |  | 1 | 2 | 3 | 4 |
|  |  |  |  |  | 4) **Mini goal:** Each member creates an action plan (what, where, when) to identify 1 resource for managing their child’s ADHD. | **Relevance** | | | |
|  |  |  |  |  |  | 1 | 2 | 3 | 4 |
|  |  |  |  |  |  | **Effectiveness** | | | |
|  |  |  |  |  |  | 1 | 2 | 3 | 4 |
|  |  |  |  |  |  | **Appropriateness** | | | |
|  |  |  |  |  |  | 1 | 2 | 3 | 4 |
|  | Additional Comments: | | | | | | | | |
|  | 4 | Modify negative beliefs about mothering and its relationship to child participation  Develop adaptive occupational identity regarding their mothering role | Social influences  Beliefs about capabilities  Role identity | Members are able to verbally de-couple their child’s ADHD behaviors from their mothering responsibility | 1) **Group Activity:**  a) Members are presented with statements/depictions that reflect the cognitive bias (i.e., mothers are to blame for child’s behavior) in various social contexts.  b) Members generate personal examples of scenarios  c) Members create alternate scenarios- role play positive reframing of belief in response to child’s behavioral challenges (Self-blame vs. reframing ADHD as a biological condition. E.g. child is not responding to parenting attempts at routinizing bedtime, mother attributes behavior to ADHD and recognizes need for additional knowledge/skills as opposed to, ‘I’m not a good enough mother to this child’) | **Relevance** | | | |
|  |  |  |  |  |  | 1 | 2 | 3 | 4 |
|  |  |  |  |  |  | **Effectiveness** | | | |
|  |  |  |  |  |  | 1 | 2 | 3 | 4 |
|  |  |  |  |  |  | **Appropriateness** | | | |
|  |  |  |  |  |  | 1 | 2 | 3 | 4 |
|  |  |  |  |  | 2) **Mini goal:** Each member identifies either an example from a personal scenario or from the media displaying cognitive bias moving from self-blame to an inquisitive stance | **Relevance** | | | |
|  |  |  |  |  |  | 1 | 2 | 3 | 4 |
|  |  |  |  |  |  | **Effectiveness** | | | |
|  |  |  |  |  |  | 1 | 2 | 3 | 4 |
|  |  |  |  |  |  | **Appropriateness** | | | |
|  |  |  |  |  |  | 1 | 2 | 3 | 4 |
| Additional Comments: | | | | | | | | | |
| Developing strategies to manage child ADHD in the school | 5 | Identify communication  barriers  Understand the gap between their child’s needs and the school’s capabilities to meet them  Increase mother advocacy skills | Knowledge  Skills  Role & Identity  Beliefs about capabilities | Members will identify a person of interest in their child’s education and be able to advocate for 1 of their child’s needs. | 1) **Short presentation:** Brief education about effective self-advocacy | **Relevance** | | | |
|  |  |  |  |  |  | 1 | 2 | 3 | 4 |
|  |  |  |  |  |  | **Effectiveness** | | | |
|  |  |  |  |  |  | 1 | 2 | 3 | 4 |
|  |  |  |  |  |  | **Appropriateness** | | | |
|  |  |  |  |  |  | 1 | 2 | 3 | 4 |
|  |  |  |  |  | 2) **Role playing:**  a) Members identify a need of their child with ADHD  b) Practice the skill of self-advocacy (multiple scenarios, different contexts) | **Relevance** | | | |
|  |  |  |  |  |  | 1 | 2 | 3 | 4 |
|  |  |  |  |  |  | **Effectiveness** | | | |
|  |  |  |  |  |  | 1 | 2 | 3 | 4 |
|  |  |  |  |  |  | **Appropriateness** | | | |
|  |  |  |  |  |  | 1 | 2 | 3 | 4 |
|  |  |  |  |  | 3) **Mini goal:** Each member identifies a communication need with another (educator, family, health provider, neighbor, Rabbi) and create an action plan to advocate for their child. | **Relevance** | | | |
|  |  |  |  |  |  | 1 | 2 | 3 | 4 |
|  |  |  |  |  |  | **Effectiveness** | | | |
|  |  |  |  |  |  | 1 | 2 | 3 | 4 |
|  |  |  |  |  |  | **Appropriateness** | | | |
|  |  |  |  |  |  | 1 | 2 | 3 | 4 |
| Additional comments: | | | | | | | | | |
| Healthy mother-healthy child:  Developing the awareness of self-care and its legitimacy | 6 | Improve engagement in health promoting activities (HPA) | Knowledge  Context and Resources  Emotion | Members will increase engagement in 1 health promoting activity of her choice | 1) **Short presentation:**  a) The parenting occupations and purposes conceptual framework (POP): Relationship between what activities parents do in order to take care of their child’s needs and what they do to improve their own capabilities so that they can care for their children.  b) Knowledge on link between healthy lifestyle behaviors and health outcomes | **Relevance** | | | |
|  |  |  |  |  |  | 1 | 2 | 3 | 4 |
|  |  |  |  |  |  | **Effectiveness** | | | |
|  |  |  |  |  |  | 1 | 2 | 3 | 4 |
|  |  |  |  |  |  | **Appropriateness** | | | |
|  |  |  |  |  |  | 1 | 2 | 3 | 4 |
|  |  |  |  |  | 2) **Group activity:**  a) Complete an activity daily time log to build awareness of personal health promoting activities (HPA) profile  b) Discussion of HPA barriers and resources | **Relevance** | | | |
|  |  |  |  |  |  | 1 | 2 | 3 | 4 |
|  |  |  |  |  |  | **Effectiveness** | | | |
|  |  |  |  |  |  | 1 | 2 | 3 | 4 |
|  |  |  |  |  |  | **Appropriateness** | | | |
|  |  |  |  |  |  | 1 | 2 | 3 | 4 |
|  |  |  |  |  | 3) **Mini goal:**  a) Each member chooses a SMART (realistic) goal* to promote their engagement in an HPA  b) Members make an action plan: visualize implementation, identify barriers, identify resources  *Therapist provides a bank of mini goals to choose from. | **Relevance** | | | |
|  |  |  |  |  |  | 1 | 2 | 3 | 4 |
|  |  |  |  |  |  | **Effectiveness** | | | |
|  |  |  |  |  |  | 1 | 2 | 3 | 4 |
|  |  |  |  |  |  | **Appropriateness** | | | |
|  |  |  |  |  |  | 1 | 2 | 3 | 4 |
| Additional comments: | | | | | | | | | |

Note: Each session is 2 hours, once a week

**Additional Comments on the whole intervention:**
